# A new dataset on product-level trade elasticities

**DOI:** 10.1016/j.dib.2022.108668

**Published:** 2022-10-12

**Authors:** Lionel Fontagné, Houssein Guimbard, Gianluca Orefice

**Affiliations:** a Banque de France, 31 rue Croix des Petits-Champs 75049 PARIS cedex 01 France, CEPII and Paris School of Economics; b CEPII, 20 avenue de Ségur 75007 Paris; c University Paris-Dauphine, PSL, Place du Maréchal de Lattre de Tassigny 75007 Paris

**Keywords:** Trade Elasticity, International Trade, Tariffs, Welfare Gains

## Abstract

This data article describes a new dataset on product-level trade elasticity, here defined as the degree of substitutability between varieties, i.e. between products exported by different countries into a given destination. The dataset contains trade elasticities for a list of more than 5000 products of the HS 6-digit classification. Trade elasticities computed using alternative sector classifications are provided as well (TIVA, GTAP, WIOD classification), by pooling the product-level observations within each sector. Starting from the prior that the coefficient associated with tariffs – a variable trade cost – corresponds to the import-demand elasticity in a standard CES structural gravity model of bilateral trade, elasticities are recovered from country-pair specific information on applied tariffs and trade. For each HS 6-digit product category we observe the universe of bilateral trade flows between countries, in value, in a given year, and the tariff (preferential or not) applied to each exporter by each importer on the specific product for 2001, 2004, 2007, 2010, 2013 and 2016. The tariff elasticity is (minus) the elasticity of substitution across products coming from different origins. Product-specific trade elasticity estimations are crucial for the evaluation of the welfare consequences of trade policies, and for the comparison of the welfare gains from trade for countries at different level of development.

**Specifications Table**

**Table:** 

Subject	Economics.
Specific subject area	Economics and International Trade*.*
Type of data	Tables and STATA file
How the data were acquired	The data reported in this article are the results of econometric estimations using STATA (econometric software) on existing data sets on international trade flows and bilateral applied tariffs data. Such existing data sets are available in the dedicated Mendeley Repository (see below).
Data format	Analyzed
Description of data collection	Our econometric estimations base on existing data sets containing information on the bilateral international trade flows among country pairs (source BACI, CEPII) and applied tariffs on imports (source MacMap, CEPII). These data sources have been selected for their high accuracy and country-product-time coverage. The econometric model used to estimate the trade elasticities reported in the present article (“*A new dataset on product-level trade elasticities*”) is the structural gravity model for trade.
Data source location	The sources of raw data used to estimate the trade elasticities are hosted at CEPII: 20 avenue de Ségur 75007 Paris, France.
Data accessibility	Raw Data available on: Mendeley repository. Repository name: Mendeley Data. Data identification number: 10.17632/8v4579gnvc.2 Direct URL to data: https://data.mendeley.com/datasets/8v4579gnvc/2 Instructions for accessing these data: no access controls, just click on the Mendeley link and the user will have access to the data.
Related research article	L. Fontagné, H. Guimbard, G. Orefice, Tariff-based product-level trade elasticities, Journal of International Economics, 137 (2022) 103593. https://doi.org/10.1016/j.jinteco.2022.103593.

**Value of the Data**

• The trade elasticity is a fundamental parameter for the evaluation of trade policies, and in particular for the calculation of the welfare change associated with various shocks of trade policies. Product-specific trade elasticities allow a more precise evaluation of such welfare changes.
• Trade elasticity estimations are employed in the analysis of cross-country transmission of macroeconomic shocks.
• All researchers, practitioners and policy makers interested in the evaluation of the welfare change from trade policy and/or the international transmission of macroeconomic shocks can directly employ the elasticities contained in the present dataset for their calculation.
• Being estimated as time-invariant parameters, product level trade elasticities can be used whenever needed for future research.

## 1 Objective

The trade elasticity estimations described in this article base on the idea that the imposition of tariffs increases the price of imports (under the assumption of full pass-through), and therefore affect the import demand. This paper adds to the related research paper (Fontagné, Guimbard and Orefice [1]) by providing a careful discussion of the empirical distribution of the product level trade elasticities and the associated database.

## 2 Data Description

The trade elasticity indicates the degree of substitutability between varieties, i.e. between products exported by different countries into a given destination. It can be recovered by (one plus) the import price elasticity, which is the extent to which the import demand of a specific product, from a specific origin, depends on its own price. In international economics and macroeconomics, this is a fundamental parameter for the evaluation of both the welfare consequences of trade policies and international transmission of macroeconomic shocks. In line with Arkolakis, Costinot and Rodrigues-Clare [2], the welfare gain from trade depends on the change in the share of domestic expenditure and the trade elasticity to variable trade costs. The trade elasticity parameter is also key for a proper evaluation of the cross-country transmission of macroeconomic shock (such as the COVID-19 induced interruption in the international supply-chain linkages). Despite the great importance of such parameter for scholars, practitioners and policy makers, there is a lack of complete and reliable estimates of product-specific trade elasticities in the previous literature.

This data article, along with the related research article (Fontagné, Guimbard and Orefice [1]), fills this gap and provides a dataset of trade elasticities for more than 5000 products of the Harmonized System (HS) 6-digit product classification. Since trade elasticities are reasonably sticky over time (i.e. almost time-invariant), the estimates contained in the present manuscript can be used and applied in quantifying the welfare consequences of trade policies, no matter the timing of the specific policy. The complete dataset on product-specific trade elasticities is made available in STATA (.dta) format, and can be accessed through a dedicated Mendeley repository [3]. Beyond the dataset on trade elasticity (“elasticity_for_publication”), we also provide in the Mendely repository all the SAS and STATA dofiles used to estimate trade elasticities (folder “STATA.zip” and “SAS.zip” respectively). A dedicated read me file in Mendeley repository will guide the user from raw data to estimated trade elasticities. The variables contained in the dataset are listed and briefly discussed in Table 1. For each product of the HS 6-digit classification we provide: (i) the trade elasticity value as it comes from estimation (i.e. the point estimate) – variable name: *epsilon_pt*, (ii) the trade elasticity value replaced by the HS 4-digit heading average when the product-specific point estimate is missing, non-significant or positive – variable name: *epsilon*, and (iii) a set of categorical variables indicating whether the original point estimate is statistically non-significant (variable name: *zero*) positive (variable name: *positive*) or missing (variable name: *missing*).

**Table 1:** Variable description.

Variable name	Type	Description
HS6	String	Product code HS 6-digit classification.
Epsilon	Number	Value of trade elasticity with replacement is non-significant, positive or missing.
Zero	Categorical (0/1)	Equal to one if the original estimate is non-significant.
Positive	Categorical (0/1)	Equal to one if the original estimate is positive and significant.
Missing	Categorical (0/1)	Equal to one if the original estimate is missing.
Epsilon_pt	Number	Value of trade elasticity without replacement (i.e. point estimate)
Positive_pt	Categorical (0/1)	Equal to one if the original point estimate is positive.

The average trade elasticity (point estimate without replacement) in our sample is -8.11, while the median value is -5.96. However, after replacing non-significant, positive or missing point estimates by average HS 4-digit specific heading, the average and median trade elasticity become respectively -10.78 and -8.04. See Table 2. Interestingly, trade elasticities differ considerably across macro HS 1-digit chapters. In Table 3 we show the summary statistics of trade elasticity (point estimate without replacement) by macro HS 1-digit chapter. For homogeneous products, such as mineral and chemical products, the average trade elasticity is high, respectively -18 and -10. It means that, considering the standardized nature of these products, the importing country can easily substitute imports across origins when a change in bilateral import price occurs. On the opposite end of the spectrum of the trade elasticity, for highly differentiated products (such as machinery and footwear) we obtain milder value of trade elasticity (respectively -6 and -3). The heterogeneity of trade elasticity across HS 6-digit products is also shown in the kernel graph reported in Figure 1 in Fontagné et al. [1]. Trade elasticities are centered around a median of -5, but very dispersed with standard deviation of 8.5. In Figure 2 in Fontagné et al. [1] we split the distribution of trade elasticity respectively for manufacturing and agriculture products.

**Table 2:** Trade elasticity summary statistics.

Variable	SD	Mean	P50	P25	P75
Epsilon_pt	8.50	-8.11	-5.96	-10.01	-3.14
Epsilon	9.43	-10.78	-8.04	-13.17	-5.18

**Table 3:** Trade elasticity summary statistics by macro HS 1-digit sector.

HS code	Description	SD	Mean	P50	P25	P75
I	Live Animals and Animal Products	9.08	-7.54	-5.15	-9.14	-2.81
II	Vegetable Products	4.55	-6.06	-5.36	-8.23	-3.04
III	Animal or vegetable fats and oils	8.69	-8.53	-6.82	-10.51	-3.68
IV	Prepared foodstuffs, beverages and tobacco	4.50	-6.17	-5.42	-8.26	-3.28
V	Mineral products	17.68	-18.50	-13.52	-26.88	-6.54
VI	Products of chemical industries	10.67	-10.33	-7.74	-13.15	-4.33
VII	Plastic and articles thereof	7.20	-8.39	-6.80	-10.58	-4.02
VIII	Raw hides and skins, leather and article thereof	4.67	-5.59	-4.51	-7.61	-1.99
IX	Wood/Cork and articles of Wood/Cork;	8.12	-8.47	-6.53	-10.73	-3.89
X	Pulp of wood or other cellulosic materials	7.42	-9.93	-9.00	-12.37	-5.10
XI	Textile and textile articles	6.86	-7.15	-5.49	-8.52	-2.81
XII	Footwear, Headgear, Umbrellas, prepared feathers	2.77	-3.61	-3.41	-4.61	-1.52
XIII	Articles of stone, plaster, ceramic and glass	4.19	-6.62	-6.48	-8.72	-3.07
XIV	Natural cultured pearls, precious stones and metals	13.52	-13.59	-10.65	-19.46	-4.00
XV	Base metals and articles of base metals	9.76	-9.59	-6.71	-11.85	-3.51
XVI	Machinery, mechanical appl., electrical machinery	5.55	-6.08	-4.74	-7.75	-2.60
XVII	Vehicles, Aircraft and transport equipment	8.53	-10.46	-7.85	-13.81	-4.58
XVIII	Optical, photographic and medical instruments	5.53	-5.61	-4.54	-7.52	-1.92
XIX	Arms and ammunitions	5.14	-6.52	-3.72	-13.30	-2.28
XX	Miscellaneous	3.42	-4.85	-4.14	-7.13	-2.01
XXI	Works of art	4.37	-5.96	-4.57	-11.20	-2.27

Note: Summary statistics based on trade elasticity values without replacement (variable epsilon_pt) but excluding positive values.

## 3 Experimental Design, Materials and Methods

Trade elasticities are obtained by estimating a structural gravity model for trade for each of the more than 5000 products of the HS 6-digit classification. In particular, we estimate the extent of the *change* in bilateral (origin-to-destination) imports value induced by a *change* in import price. In our approach we instrument the *change* in the bilateral import price with *changes* in bilateral applied tariffs.^1^ In a structural gravity model for trade having the value of bilateral imports as dependent variable, the coefficient (i.e. point estimate) associated to the bilateral applied tariff represents the trade elasticity.^2^ Potential confounding factors in estimations, such as the bilateral transport costs or common cultural traits between importer and exporter countries are controlled by the inclusion of country-pair specific control variables: (i) distance (in Km), (ii) common language, (iii) common border and (iv) common colonial relationship. Country-pair controls are from CEPII gravity dataset [4]. Any country-product specific demand (supply) shock at importing (exporting) country is fully captured by country-year specific categorical variables (or fixed effects in the econometrics jargon). Please refer to Fontagne et al. [1] for a more detailed discussion on the estimation's methodology.

Our estimates of trade elasticities are therefore based on two primary sources of data: bilateral product-specific import values and applied tariffs. Bilateral import data are from CEPII BACI data [5]. For the full matrix of importer and exporter countries in the world, the BACI database provides information on bilateral import flows (Free On Board, FOB), in current US dollars, during the period 1996-2016, at the HS 6-digit level. Imports value data are then combined with data on bilateral applied tariffs for all importers and exporters and all products. This information is provided by MAcMap-HS6 CEPII dataset [6]. For each product and an almost exhaustive list of country pairs in the world, MAcMap-HS6 provides the preferential applied tariff the years 2001, 2004, 2007, 2010, 2013 and 2016. In terms of country coverage, on the importer side we are constrained by the 2010 release of MAcMap-HS6, providing applied tariff data for a set of 152 importers. On the exporting side the constraint is less binding and we keep all the exporting countries present in BACI since 2001. Ultimately, we have 189 exporters to 152 destinations in each year. After merging the BACI and MAcMap-HS6 datasets, for each of the 5,052 HS 6-digit product, we end up with a panel dataset of country pairs for the years 2001, 2004, 2007, 2010, 2013 and 2016.

With the product-specific panel datasets at hands, we finally estimate the structural gravity model for trade for each of the 5,052 HS 6-digit products and extract the point estimates and the standard errors associated to the bilateral applies tariffs variable. In the final dataset we provide the trade elasticity with replacement (when point estimate is non-significant, positive of missing) for an easy plug-and-play use, as well as the original point estimates (as they come from estimates) for the interested user.

## Ethics Statements

Our work did not involved human subject, nor animal experiment or collection from social media platforms.

## CRediT Author Statement

**Lionel Fontagné:** Conceptualization, Methodology, Software; **Houssein Guimbard:** Conceptualization, Methodology, Software; **Gianluca Orefice:** Conceptualization, Methodology, Software.

## Declaration of Competing Interest

The authors declare that they have no known competing financial interests or personal relationships that could have appeared to influence the work reported in this paper.

## Data Availability

Trade Elasticity - Data in Brief (Original data) (Mendeley Data).

## Footnotes

1 We implicitly assume that a change in tariffs is fully passed into import price (i.e. full pass-through assumption).

2 Strictly speaking the point estimate (in absolute value) associated to the bilateral applied tariff represents the elasticity of substitution parameter σ that can be transformed into trade elasticity as 1-σ.
